# Modeling and Analysis of HIV-1 Pol Polyprotein as a Case Study for Predicting Large Polyprotein Structures

**DOI:** 10.3390/ijms25031809

**Published:** 2024-02-02

**Authors:** Ming Hao, Tomozumi Imamichi, Weizhong Chang

**Affiliations:** Laboratory of Human Retrovirology and Immunoinformatics, Frederick National Laboratory for Cancer Research, Frederick, MD 21702, USA; ming.hao@nih.gov (M.H.); timamichi@mail.nih.gov (T.I.)

**Keywords:** HIV-1 Pol structure, immature protein structure modeling, computational structural biology, domain assembly, loop modeling, homology modeling

## Abstract

Acquired immunodeficiency syndrome (AIDS) is caused by human immunodeficiency virus (HIV). HIV protease, reverse transcriptase, and integrase are targets of current drugs to treat the disease. However, anti-viral drug-resistant strains have emerged quickly due to the high mutation rate of the virus, leading to the demand for the development of new drugs. One attractive target is Gag-Pol polyprotein, which plays a key role in the life cycle of HIV. Recently, we found that a combination of M50I and V151I mutations in HIV-1 integrase can suppress virus release and inhibit the initiation of Gag-Pol autoprocessing and maturation without interfering with the dimerization of Gag-Pol. Additional mutations in integrase or RNase H domain in reverse transcriptase can compensate for the defect. However, the molecular mechanism is unknown. There is no tertiary structure of the full-length HIV-1 Pol protein available for further study. Therefore, we developed a workflow to predict the tertiary structure of HIV-1 NL4.3 Pol polyprotein. The modeled structure has comparable quality compared with the recently published partial HIV-1 Pol structure (PDB ID: 7SJX). Our HIV-1 NL4.3 Pol dimer model is the first full-length Pol tertiary structure. It can provide a structural platform for studying the autoprocessing mechanism of HIV-1 Pol and for developing new potent drugs. Moreover, the workflow can be used to predict other large protein structures that cannot be resolved via conventional experimental methods.

## 1. Introduction

With advances in molecular biological technologies, protein amino acid sequences can be obtained quickly from DNA sequence analysis using routine procedures, and the secondary and tertiary structures of many proteins have also been resolved and are available in a publicly available database, RCSB PDB [1]. The main method used with experimental techniques to decipher the secondary and tertiary structures of proteins is X-ray crystallography [2,3]. However, many proteins are not soluble or stable after they are purified, and not all of them can be crystalized even if the researchers can obtain stable, purified proteins in solution using various techniques [4,5,6,7]. Another popular method to solve the protein structure is Cryogenic Electron Microscopy (cryo-EM), a cryomicroscopy technique applied to a sample cooled to a cryogenic temperature, which can preserve the biological specimen structure by embedding it in an environment of vitreous ice [8,9,10]. With advances in sensitivity and resolution, it is widely used as an alternative to X-ray crystallography to solve the protein structure [11,12]. The advantage of cryo-EM is that there is no need to crystalize the protein. However, it still could not resolve the structures of many proteins. For example, Harrison et al. [13] published the protease (PR) and reverse transcriptase (RT) components of human immunodeficiency virus type-1 (HIV-1) Pol polyprotein using the cryo-EM method using in vitro expressed full-length Pol polyprotein. Integrase (IN) is largely not observed in the structures except for the first 49 amino acids in the N-terminal domain (NTD) of IN identified via focused classification in the region expected. The authors suggested that the cause is flexibility between RT and IN, and within the subdomains of IN [13].

HIV-1 is the virus that causes acquired immunodeficiency syndrome (AIDS), a global epidemic. Despite decades of research, there is no cure for the disease, even though several classes of anti-viral drugs targeting HIV proteins, such as RT, PR, and IN, have been developed. Variants resistant to the drugs develop quickly in patients after treatment, leading to the need to change to a regimen with a new set of drugs. Thus, there is an urgent demand to design new drugs. Most currently available drugs are designed to act against mature forms of HIV-1 proteins: PR, RT, and IN. PR, RT, and IN are initially translated from a genomic HIV RNA as a single-chain polyprotein along with Gag proteins, Gag-Pol (MW 160 kDa). During the maturation process, the polyproteins dimerize and are then cleaved by the embedded PR [14,15,16,17] to produce each mature protein. A new class of drugs targeting dimerization/maturation has been investigated [18,19]. However, due to a lack of detailed structure models of full-length dimerized polyproteins, the molecular mechanisms of inhibition are still not fully understood. One major reason for this is that the tertiary structure of HIV-1 Gag-Pol polyprotein is unknown, partly due to the large size of the protein, the conformational flexibility among domains, and the limitations of current technologies, such as those described above. Our recent studies demonstrated that the combination of M50I and V150I polymorphic mutations in IN (IN:M50I/V151I) increased the dimerization of Gag-Pol and inhibited the initiation of autoprocessing of the Gag-Pol polyprotein, subsequently suppressing viral infection/replication and reduced virus release from HIV-infected cells [20,21]. The defect caused by IN:M50I/V151I could be compensated for by a third mutation, S17N in IN [20,22] or N79S in RNase H (RH) [22]. We further demonstrated that the C-terminal domain of RH and the C-terminus amino acid residue in IN regulate virus release and the autoprocessing of defective HIV-1 processing with IN:M50I/V151I [22]. Although we have modeled the tertiary immature structures of full-length IN and RH + IN [20,21] and investigated them using different methods and related tools (PremPS, DynaMut2 and DDMut) for stability prediction upon mutation [23,24,25], the effects of these mutations on autoprocessing are not fully understood. A similar phenomenon was observed in another group of mutations (IN:T124N/V165I/T174I) that emerged in cell culture in the presence of pyridine-based multimerization-selective HIV-1 integrase inhibitors (MINIs), KF1116. Double mutations (IN:T124N/T174I) inhibited the proteolytic processing of HIV-1 polyproteins Gag and Gag-Pol, resulting in immature virions. IN:V65I could partially compensate for the defect [26]. Only the mechanism of the resistance to KF116 could be explained by the IN structure with these substitutions. To understand the mechanism of these mutations’ effects on Gag-Pol processing, we needed the tertiary structure of full-length Gag-Pol or Pol to investigate the interactions between these mutations in the unprocessed (immature) structural context. We decided to build the structure computationally to obtain a full-length HIV-1 Pol tertiary structure.

In this study, we developed a novel workflow to predict the tertiary structure of HIV-1 Pol from the HIV-1 NL4.3 strain [27], which is a laboratory-adapted strain used in many laboratories, including our laboratory. Our HIV-1 Pol structure was built upon the published partial HIV-1 Pol structure mentioned above [13] with the addition of other immature domains modeled with relevant experimentally determined structures obtained from a publicly available database [1]. This HIV-1 Pol dimer structure is the first full-length HIV Pol tertiary structure and would provide researchers with a great base to investigate Gag-Pol autoprocessing and develop novel anti-HIV drugs. The workflow developed in this study may be used to solve other large polyproteins such as Gag/Pol/Gag-Pol protein from other retroviruses (e.g., human foamy virus (HFV), Moloney murine leukemia virus (MoMLV), feline immunodeficiency virus (FIV), mouse mammary tumor virus (MMTV), human T-lymphotropic virus (HTLV), and respiratory syncytial virus (RSV)) [28] and ORF1a/ORF1ab from coronaviruses (e.g., severe acute respiratory syndrome coronavirus (SARS-CoV), Middle East respiratory syndrome coronavirus (MERS-CoV), and severe acute respiratory syndrome coronavirus (SARS-CoV2)) [29].

## 2. Results

### 2.1. Overview of the Workflow to Predict the Structure of the HIV-1 Pol Polyprotein

**Figure 1:**
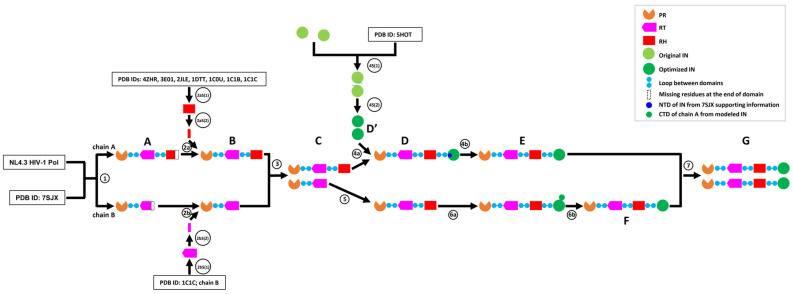
Flowchart of HIV-1 NL4.3 Pol dimer structure prediction. Step 1: part of the Pol structure with missing fragments for both chain A and chain B can be predicted based on the reference structure of 7SJX. Steps 2a and 2b: the missing fragments from step 1 can be fixed based on the template-based modeling methods and added to the structure from step 1. Step 3: part of the Pol dimer can be predicted based on the anchor-based method. Steps 4s(1) and 4s(2): IN dimer can be predicted based on the anchor-based methods and the dimer is further optimized with SWISS-MODEL. Steps 4a and 4b: one IN subunit can be assembled into chain A of Pol based on the known IN NTD position. Step 5: RH domain can be assembled into chain B of Pol. Steps 6a and 6b: IN domain can be assembled into chain B of Pol based on the empirical strategy. Step 7: the final NL4.3 Pol dimer can be modeled after large-scale optimization and refinement.

In this study, we developed an integrated computational workflow (Figure 1) to build an HIV-1 NL4.3 Pol dimer structure by using a set of protein structure analysis web applications and software tools (Table 1). Briefly, using HIV-1 NL4.3 Pol sequence and published chain A (7sjxA) and chain B (7sjxB) of the partial HIV-1 Pol structure (PDB ID: 7SJX) as inputs, we modeled chain A and chain B structures of partial NL4.3 Pol with SWISS-MODEL [30] (step 1). The missing regions within the existing structure were modeled at this step. To add the seven missing amino acids (aa) at the C-terminus of RH in chain A, we first modeled the full-length RH structure with Robetta [31,32] (step 2aS(1)) because the structure for the last seven aa in RH had not been resolved in any structure in the PDB database. We then extracted the last seven aa fragment structures (step 2aS(2)) and added them to NL4.3 Pol chain A using PyMOL (v.2.5.0), followed by the optimization of the chain A structure with SWISS-MODEL (step 2a). Similarly, the missing 12 aa fragment structure at the C-terminus of RT in chain B was added and optimized with the same procedure using the published structure (PDB ID: 1C1C) (step 2bS(1), 2bS(2) and step 2b). The dimer of the partial NL4.3 chain A and chain B of Pol was then assembled with the anchor-based method using the dimer 7SJX as the anchor (step 3). The IN dimer was also modeled with the anchor-based method (step 4S(1)) and optimized with GalaxyRefineComplex [33] (step 4S(2)). One IN subunit from the optimized dimer was added to chain A using GalaxyDomDock [34] (step 4a) and optimized with SWISS-MODEL (step 4b). Similarly, the RH domain was added to chain B using GalaxyDomDock and optimized with SWISS-MODEL (step 5). Finally, we added the IN subunit to the C-terminus of RH in the NL4.3 chain B of Pol with SWISS-MODEL (step 6a, 6b). In this step, we attached 59 aa IN (C-terminus domain) CTD from chain A to the target IN C-terminus, which made it possible to build the full NL4.3 chain B of Pol to be compatible with chain A. The full-length NL4.3 chain A and chain B of Pol were modeled to a dimer with the anchor-based method using 7SJX as the anchor and optimized with YASARA [35], followed by optimization with SWISS-MODEL (step 7).

### 2.2. Comparing the Structure of Mature HIV-1 Proteins with Corresponding Segments in HIV-1 Pol Polyprotein

**Figure 2:**
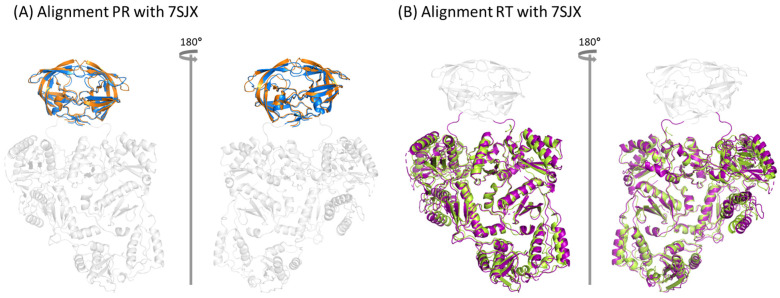
Structure comparison between mature and immature proteins. (**A**) Mature PR structure (PDB ID: 2HB4) aligned to 7SJX, where 2HB4 is colored in orange and the PR domain of 7SJX is colored in marine with rest of the structure colored in gray. (**B**) Mature RT structure (PDB ID: 1DLO) aligned to 7SJX, where 1DLO is colored in limon and the RT domain of 7SJX is colored in purple with rest of the structure colored in gray.

The structures of the mature HIV-1 proteins originating from the Pol protein have been resolved by many groups [38,39,40,41,42,43,44,45,46,47,48,49,50]. However, the structures of these proteins in unprocessed Gag-Pol context are mostly unknown. Recently, the PR and RT components in the Pol polyprotein have been resolved with cryo-EM using expressed HIV-1 Pol polyprotein [13]. To evaluate the differences between structures of PR and RT within the unprocessed Pol polyprotein (immature structure) and mature PR and RT structures, we aligned the mature PR structure with PDB ID 2HB4 (Figure 2A), and the mature RT structure with PDB ID 1DLO (Figure 2B), to their immature counterparts from the structure with PDB ID 7SJX using PyMOL. The results show that the mature protein structures were very close to their unprocessed counterparts in the Pol polyprotein, except for the residues either at the end of each domain or in the loop region. Based on this observation, we built other components in the Pol polyprotein based on the corresponding mature protein structures.

### 2.3. Comparison between NL4.3 HIV-1 Pol Sequence and That Extracted from the Crystalized Pol Structure

**Figure 3:**
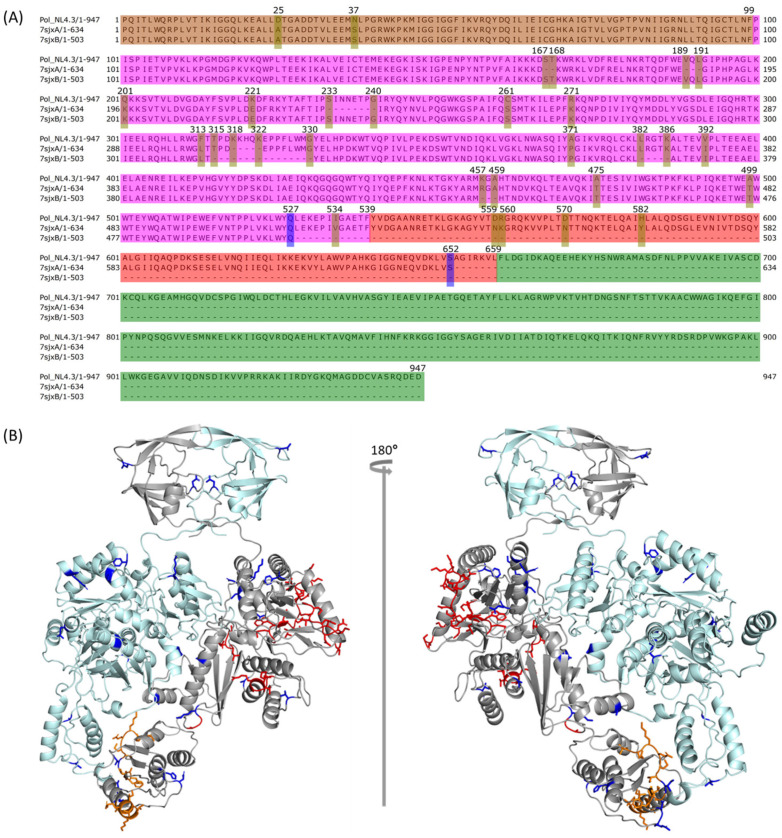
The predicted structure of the PR-RT component in HIV-1 NL4.3 Pol polyprotein. (**A**) Sequence comparison between HIV-1 NL4.3 Pol and those extracted from 7SJX (chain A: 7sjxA; chain B: 7sjxB), where mutations and missing fragments in the middle are highlighted with a yellow bar, and the last determined positions for both 7sjxA and 7sjxB are highlighted with a blue bar. The PR domain, RT domain, RH domain and IN domain are colored in orange, magenta, red, and green, respectively. (**B**) Modeled part of NL4.3 Pol dimer consisting of PR + RT + RH in chain A and PR + RT in chain B where chain A is colored in gray and chain B in pale cyan. The modeled mutations, missing fragments from either the middle or the end of sequence are colored in blue, red, and orange, separately.

To predict the full-length HIV-1 strain NL4.3 Pol structure, we leveraged the published partial HIV-1 Pol polyprotein structure, 7SJX [13]. The HIV-1 NL4.3 Pol polyprotein contains four domains: PR, RT, RH, and IN, with a total of 947 aa. We compared the complete NL4.3 HIV-1 Pol protein sequence with those from 7sjxA and 7sjxB by using T-Coffee implemented in Jalview [36,37] (Figure 3A). The entire IN structure cannot be determined in the structure of 7SJX. The entire RH is also missing in chain B (7sjxB), which is common for most known RT heterodimer structures [38,39,41]. In addition, 7SJX was also missing several fragments in RT and RH domains. There were also some amino acid differences between the NL4.3 HIV-1 Pol sequence and that from the 7SJX structure. To predict the complete HIV-1 NL4.3 Pol structure, we first needed to model several Pol structure components, including the whole domain of IN for both chain A and chain B, the whole RH domain for chain B, the missing fragments existing either in the middle of the RT domain or at the end of the RH domain of chain A and the RT domain of chain B, and the different amino acids.

### 2.4. Modeling PR + RT + RH for Chain A and PR + RT for Chain B of Pol

Since the published HIV-1 polyprotein, 7SJX [13], is the only known 3D structure with the PR and RT components of Pol, we used this structure as the starting point to build the complete Pol polyprotein structure of HIV-1 strain NL4.3. As described above, the published HIV-1 Pol structure contains PR + RT domains: chain A has PR, RT, and most RH regions, with seven aa missing at the C-terminus of RH (Figure 3A); chain B has PR and RT regions, with twelve aa missing at the C-terminus of RT (Figure 3A). We used the chain A and chain B structures of 7SJX (7sjxA and 7sjxB) independently to guide SWISS-MODEL [30] to model the structure of the corresponding regions of chain A and chain B for HIV-1 strain NL4.3 Pol (Figure 1 step 1 and structure A). The last seven residues (positions: 653–659) at the C-terminal end of the RH domain in chain A and the last 12 residues at the C-terminal end of RT in chain B are missing due to a lack of coordinates in the template of 7sjxA and 7sjxB.

To obtain the structure of the seven missing amino acids at the C-terminus of RH in Pol chain A, we first employed Robetta [31,32] to model the whole RT p66 structure of chain A with 560 aa using multiple templates (i.e., 4ZHR [38], 3E01 [39], 2JLE [40], 1DTT [41], 1C0U [42], 1C1B [43], and 1C1C [43]) with default options. These seven template structures are all DNA-free crystal structures. We expected these structures to be more similar to the immature conformations than those structures containing DNA. They exhibit high homology with the sequence of HIV-1 NL4.3 Pol RT p66 domain, ranging from 93% to 98%. Finally, we determined the optimal model by using 3E01 as the reference structure due to its completeness in the CTD domain compared to other templates. The complete RT p66 structure was then aligned with the HIV-1 NL4.3 Pol chain A incomplete RT + RH domain from step 1 (Figure 1 structure A chain A) using PyMOL (v.2.5.0). We then used the PyMOL “alter” and “create” commands to incorporate the last seven aa of modeled RH into the missing part of RH in chain A of Pol and optimized the merged structure with SWISS-MODEL. As a result, we obtained the complete structure with PR, RT, and RH (Figure 1 step 2a, structure B chain A). Similarly, we used the same process to generate the complete RT for chain B. We selected the published RT structure (PDB ID: 1C1C) as the optimal template, since it had higher identity (93.12%) with the RT reference sequence. We then merged the last 12 aa of the chain B structure from 1C1C with the HIV-1 NL4.3 Pol chain B structure obtained from the previous step (Figure 1 step 1, structure A chain B) to obtain the complete RT in chain B of Pol (Figure 1 step 2b, structure B chain B).

To eliminate any conflict caused by the changes in chain A and chain B of HIV-1 NL4.3 Pol that we obtained from the previous step (Figure 1 structure B), we assembled them into the dimer using the anchor-based method and then optimized with SWISS-MODEL (Figure 1 step 3 and structure C, Figure 3B) using the published partial Pol structure 7SJX as the anchor. This dimer also served as the mapping target for growing chain A and chain B in the following steps to make sure that no conflicts were created.

### 2.5. Assembly of PR + RT + RH with IN in Chain A of Pol

**Figure 4:**
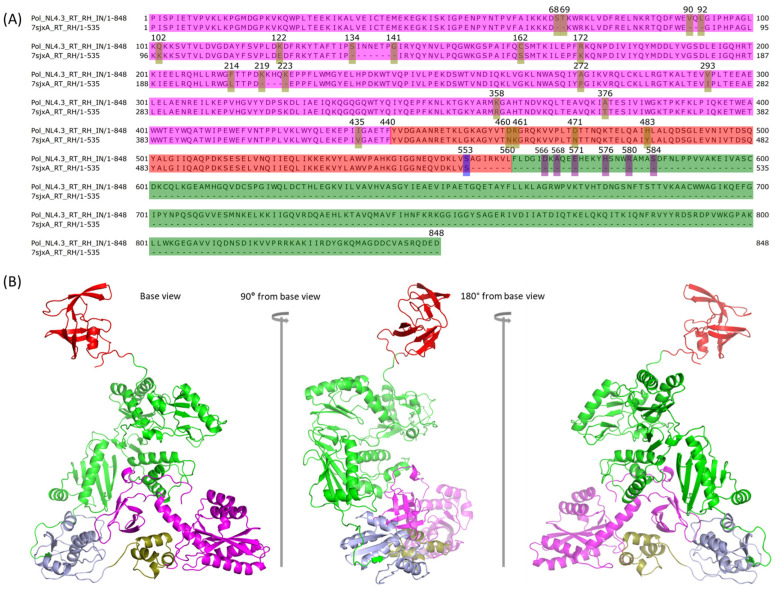
The predicted structure of the full-length HIV-1 NL4.3 Pol chain A. (**A**) Sequence comparison between RT + RH + IN of HIV-1 NL4.3 Pol and RT + RH of 7sjxA, where mutations and missing fragments in the middle are highlighted with a yellow bar, the last determined position for 7sjxA is highlighted with a blue bar, and the tested starting positions for assembly from the IN domain are highlighted with a purple bar. The RT domain, RH domain, and IN domain are colored in magenta, red, and green, respectively. (**B**) Modeled chain A of NL4.3 HIV-1 Pol where the PR domain is colored in red, RT domain in green, RH domain in light blue, and IN domain in magenta with the first 49 residues are highlighted with a deep olive color.

We have previously modeled the HIV-1 NL4.3 IN monomer [21], which was built upon eight DNA-free partial IN structures with the assumption that they were closer to the immature IN structure. Since we wanted to build the NL4.3 Pol dimer, we needed to assemble the IN dimer first. We used the anchor-based method to model the IN dimer using the IN structure of 5HOT as the anchor (Figure 1 step 4S(1)). 5HOT is a homodimer and DNA-free crystal structure of partial IN. The dimer was further optimized by GalaxyRefineComplex [34] (Figure 1 step 4S(2), Figure 1 structure D′). GalaxyDomDock was then used to perform the domain assembly of PR + RT + RH with NL4.3 IN. We used the “1-linker case” option and “domain 1” as the structure of NL4.3 RT and RH in chain A to comply with the domain length limitation of 600 aa and “domain 2” as NL4.3 IN. For the domain length selection, we selected the last experimentally determined amino residue, S553, of RH from 7sjxA as the last position in the first domain (positions: 1–553) (Figure 4A). For the second domain of IN, we experimented with different sequence ranges from different starting positions: 566 (linker length: 12), 568 (linker length: 14), 571 (linker length: 17), 576 (linker length: 22), 580 (linker length: 26), and 584 (linker length: 30), respectively. The end position was 848 (Figure 4A). As a result, we obtained 300 linker prediction models because we set GalaxyDomDock to return the top 50 models for each input set. Given the large number of linker models, the determination of the most optimal one was a challenging task. Herein, we adopted a knowledge-based method to select the proper model after removing those models with apparent conflicts with existing structures from either chain A or chain B after mapping the corresponding model to the modeled Pol dimer above (Figure 1 structure C). Even though the IN structure could not be resolved, the relative position of the first 49 residues of IN NTD to RT in Pol polyprotein was determined in the publication by Harrison et al. using focused classification on IN density extending from the RT p66-like portion of Pol [13]. We used this structure as a reference to identify the optimal model from GalaxyDomDock. The model_30 from the “domain 1: 1–553” and “domain 2: 566–848” option was identified as the best model. We connected PR back to RT + RH + IN using PyMOL (Figure 1 step 4a, structure D chain A), and the full-length NL4.3 Pol chain A was then optimized with SWISS-MODEL (Figure 1 step 4b). It was then mapped to the partial Pol dimer obtained in the previous step (Figure 3B) to make sure it was free of conflicts (Figure 1 structure E chain A, Figure 4B).

### 2.6. Assembly of RT with RH in Chain B of Pol

**Figure 5:**
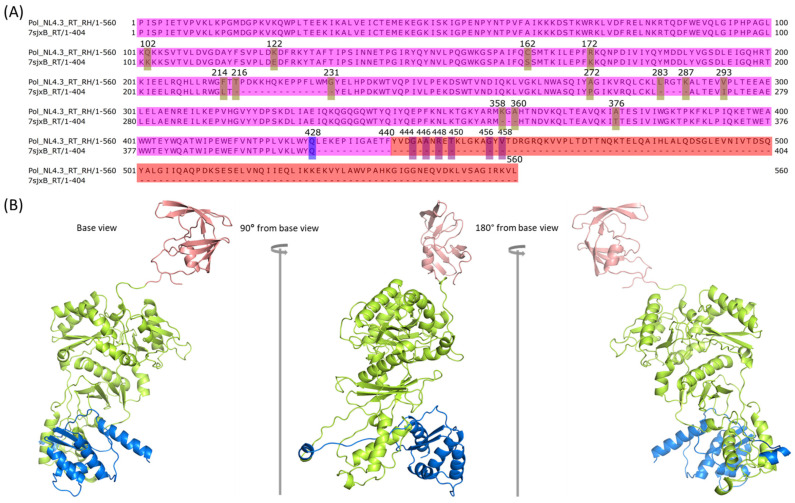
The predicted structure of the full-length HIV-1 NL4.3 Pol chain B. (**A**) Sequence comparison between RT + RH of NL4.3 HIV-1 Pol and RT of 7sjxB, where mutations and missing fragments in the middle are highlighted with a yellow bar, the last determined position for 7sjxB is highlighted with a blue bar, and the tested starting positions for assembly from the RH domain are highlighted with a purple bar. The RT domain, RH domain, and IN domain are colored in magenta, red, and green, respectively. (**B**) Modeled part of chain B in NL4.3 HIV-1 Pol where the PR domain is colored in salmon pink, the RT domain in lemon, and the RH domain in blue.

We employed GalaxyDomDock to assemble RH into RT, which was extracted from the modeled PR + RT of chain B (Figure 1 step 5). The option “domain 1” was set as the NL4.3 HIV-1 RT domain of chain B from modeled Pol (1–428, 428th residue is the last residue in the experimentally determined structure, 7SJX), and “domain 2” was set as the RH of chain B, tested with five different starting positions with different linker/loop lengths between RT and RH in chain B: 444 (linker length: 15), 446 (linker length: 17), 448 (linker length: 19), 450 (linker length: 21), 456 (linker length: 26), and 458 (linker length: 28) (Figure 5A). We selected the optimal model to ensure that (1) no conflict existed between the modeled RT + RH with either chain A or chain B, and (2) CTD from the new assembled RH should be directed towards the NTD of IN from chain B, which was obtained by mapping the IN dimer into the modeled chain A of IN. As a result, we selected model_5 of the top 50 models from the “domain 1: 1–428” and “domain 2: 456–560” combination as the best model. However, when carefully inspecting the optimal model in PyMOL, we found that a dashed line existed from residues 453 to 455, which may have been caused by the returned model from GalaxyDomDock, internally. To fix this issue, we used the optimal model as a template to perform homology modeling using SWISS-MODEL to make sure that all fragments of the model were reasonable. After that, we assembled PR back to the above modeled RT + RH, followed by SWISS-MODEL optimization (Figure 1 step 5). At this point, we finished the PR + RT + RH domain assembly for chain B of NL4.3 HIV-1 Pol (Figure 1 structure D chain B, Figure 5B).

### 2.7. Assembly of RH with IN in Chain B of Pol

Next, we needed to model the last linker/loop between the RH CTD and IN NTD of chain B in NL4.3 Pol, which is the most challenging task given that the relative conformations between RH and IN for chain B have already been fixed, leading to limited structural space for modeling. We employed SWISS-MODEL instead of GalaxyDomDock to deal with this challenge. However, we could not obtain an NL4.3 Pol chain B structure which did not conflict with chain A of the Pol dimer using IN alone. The last 59 residues in the region SRDPVWKGPAKLLWKGEGAVVIQDNSDIKVVPRRKAKIIRDYGKQMAGDDCVASRQDED in IN CTD (IN_CTD_59_A) (Figure 3) of chain A were found to be the main conflicting section. To solve this problem, we extracted IN_CTD_59_A and modeled it as the part of IN CTD of chain B by using the “create” command implemented in PyMOL. We obtained the “extended” structure (a total of 1006 aa from PR:99aa + RT:440aa + RH:120aa + IN:288aa + IN_CTD_59_A:59aa) in PDB format (Supplementary Figure S1A). We then removed IN_CTD_59_A and optimized the structure with SWISS-MODEL (Supplementary Figure S1B). Most of the conflicting sections were resolved with one position conflicting with the loop/loop intersection near the RH CTD of chain B (Supplementary Figure S1C,D). We hypothesized that this could be caused by the wrong length of the linker. Therefore, we performed more experiments by adding more residues from chain B RH CTD to the linker/loop sequence. We investigated four options in total by adding one residue (V at 651), two residues (LV at 650, 651), three residues (KLV at 649–651), and four residues (DKVL at 648–651) (Figure 3A), respectively. We found that the homology model from SWISS-MODEL with the “two residues (LV)” option had no conflict with the existing modeled structures (Figure 1 step 6a, structure E chain B). We then employed SWISS-MODEL once again to perform optimization for the full-length chain B of NL4.3 Pol after removing the additional 59 aa IN CTD from chain A and obtained the full-length chain B of HIV-1 NL4.3 Pol (Figure 1 step 6b, structure F).

### 2.8. Complete Pol Dimer Construction and Optimization

**Figure 6:**
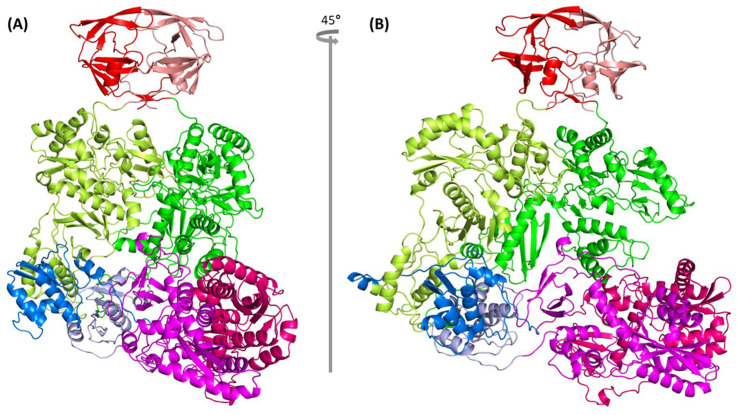
Predicted HIV-1 NL4.3 Pol dimer structure. (**A**) Front view; (**B**) 45° rotation along y-axis from (**A**) where the PR domain is colored in red, the RT domain is green, RH is light blue and IN is magenta for chain A, while for chain B, the same domains as chain A are colored in salmon pink, lemon, blue, and hot pink, respectively.

We used the anchor-based method to assemble the homodimer Pol of full-length chain A and chain B with the published partial HIV Pol structure, 7SJX, as the anchor (Figure 1 step 7). The dimer structure was optimized with the YASARA Energy Minimization Server [35]. The optimized structure was further optimized with SWISS-MODEL to obtain the final HIV-1 strain NL4.3 Pol dimer structure (Figure 1 structure G, Figure 6) and its PDB file can be obtained online (Supplementary Data). The complete Pol structure does not exhibit a linear form, but rather exhibits an “L” shape where the IN dimer is positioned at the bottom of the “L”. To further evaluate the model stability, we performed the molecular dynamics (MD) using GROMACS (version 2021) [51]. We used all-atom OPLS force field and the SPC/E water as the solvent. We performed 50,000 steps for energy minimization, and 300 ps equilibration for both NVT and NPT. Finally, we ran a 5 ns production MD simulation for investigating structural stability. Other parameter settings follow the protein in a water simulation condition [52]. The results showed that the root mean square deviation (RMSD) balanced at ~0.35 nm, indicating that the structure is stable (Supplementary Figure S2). We also compared the originally modeled HIV-1 Pol dimer with the representative structures from 4.8 ns, 4.9 ns, and 5 ns. The result indicated that all four structures aligned well with the average RMSD of ~3.032 Å based on the PyMOL align operation (Supplementary Figure S3).

### 2.9. Quality Measure of Modeled Pol Dimer

To evaluate the quality of the HIV-1 NL4.3 Pol model generated in this study, we first used MolProbity [53]. The MolProbity score is a combined score with a lower value indicating better model quality [53]. The MolProbity score for the published HIV-1 partial Pol 7SJX was 2.93, while the MolProbity scores of the YASARA-optimized and SWISS-MODEL optimized HIV-1 NL4.3 Pol structures were 3.16 and 1.01, respectively, indicating that our final HIV-1 NL4.3 Pol structure, after SWISS-MODEL optimization, is of good quality and that SWISS-MODEL can be used as a homodimer refinement tool in addition to its main function of homology modeling.

**Figure 7:**
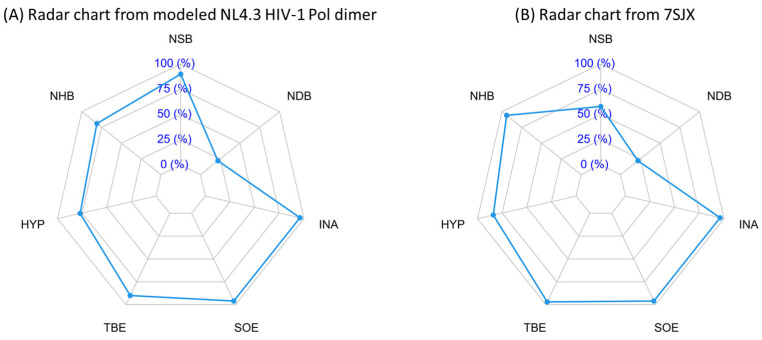
Comparison of PISA radar chart between HIV-1 NL4.3 Pol dimer and published partial HIV Pol dimer. (**A**) Radar chart from modeled NL4.3 HIV-1 Pol dimer, where NSB refers to the number of salt bridges, NHB refers to the number of hydrogen bonds, HYP refers to hydrophobic *p*-value, TBE refers to total binding energy, SOE refers to solvation energy, INA refers to interface area, and NDB refers to the number of disulfide bonds. If most points exceed the 50% threshold, it indicates that the interface is likely to be significant for biological assembly. Otherwise, interfaces with radar area, fitting within the 50% threshold, are more likely to be artefacts of crystal packing. (**B**) Radar chart from reference structure of 7SJX.

Second, we used the PISA radar plot [54] to assess our modeled HIV-1 NL4.3 Pol dimer (Table 2). The PISA radar plot is used to indicate whether the assembly for the dimer structure is biologically relevant by investigating the interactions from the interface. For the radar area, if most points exceed the 50% threshold, it indicates that the interface is likely to be significant for biological assembly. As shown in Table 2 and Figure 7, the HIV-1 NL4.3 Pol dimer structure shows six of seven points >50%, indicating that the complete Pol dimer model is biologically meaningful. We also noticed that 7SJX, as a control, shows a similar trend (Figure 7).

## 3. Discussion

Autoprocessing is the first step to release embedded PR in the HIV-1 Gag-Pol polyprotein through its processing, which requires the homodimerization of the Gag-Pol. This step plays a critical role in virus maturation. Two different combinations of double amino acid substitutions, IN:M50I/V151I [20,21,22] and IN:T124N/T174I [26], were found to inhibit autoprocessing and inhibit virus replication and release. While IN:T124N/T174I could be partially compensated by V165I substitution in IN, IN:M50I/V151I could be restored by S17N substitution or D288 substitutions in IN, or N79S substitution or a deletion of the C-terminal domain of RH, suggesting that these substitutions or deletions affect the proteolytic processing by PR. However, the mechanism is not fully understood and there is no structure available to study this in the polyprotein context. Therefore, we built the HIV-1 Pol homodimer structure which includes PR, RT, RH, and IN, making it possible to study the interaction between the substitutions in IN and PR as well as the substitutions and deletion in RH.

To build a full-length HIV-1 Pol structure, we first tried the popular tool AlphaFold2 [55,56]. When we used 7SJX [13] as the reference structure, we found that the structure (monomer) predicted by AlphaFold2 exhibited large differences in some parts compared with 7SJX. In addition, some parts of the predicted structure showed clashes with chain B of 7SJX (Supplementary Figure S4A) when we aligned the AlphaFold2 structure to chain A of 7SJX. It was the same case (showing clashes with chain A) when we aligned it to chain B of 7SJX (Supplementary Figure S4B). Therefore, we developed a computational workflow which successfully predicted a dimer structure of HIV-1 NL4.3 Pol polyprotein with a set of different software tools and web applications. The structure was built upon the longest known published partial tertial HIV-1 Pol structure, which contains PR and a partial RT heterodimer (RTp66/RTp51) even though the expressed protein used in the study contains full-length Pol [13]. The advantage to predict the full-length HIV Pol structure is that we can obtain the structure of the wild-type sequence. On the other hand, some amino acids in the protein sequence are sometimes substituted to meet the requirements of high protein concentration and long-term stability for the protein structural study [57,58,59].

The quality of the returned HIV-1 NL4.3 Pol structure was evaluated by MolProbity and PISA radar interface parameters. Evaluation results indicate that our final HIV-1 Pol dimer model is comparable to or better than the published partial HIV-1 Pol structure [13]. Since the assembly of the RH-IN linker was referenced to the 49 aa NTD IN structure which was obtained with focused classification in the expected region [13], our model will only represent a portion of the Pol dimer structure. Moreover, since the structure was obtained based on computational prediction, it needs to be further confirmed by wet lab evidence. Nevertheless, this structure is the first tertiary structure of HIV-1 Pol polyprotein, which includes PR, RT, RH, and IN. The structure makes it possible to investigate these enzymes in the immature conformation and how the substitution of the residue in IN or RH affects the structure and activity of PR in the same molecule. The structure can also be used to study the interaction of potential drug candidates with the HIV-1 polyprotein and the effect of these drug candidates on the maturation of these enzymes. We have performed docking analysis with a PR inhibitor (darunavir), RT inhibitor (GW695634), and IN inhibitor (KF116) with our NL4.3 HIV-1 Pol dimer. The detailed methods can be found in the Supplementary Information. The results indicated that the PR component in HIV-1 Pol can readily bind to the PR inhibitor, darunavir (Supplementary Figure S5A,B). However, for RT and IN inhibitors, they cannot bind directly to the RT and IN components of our NL4.3 HIV-1 Pol dimer since the binding pocket conformations are different from those of the reference PDB structures. Therefore, we performed an additional step compared to the PR inhibitor, which is structural optimization preprocessing by YASARA for the aligned complex before running the docking analysis. After the optimization, the RT and IN inhibitors can be predicted by the docking tool (gnina [60,61]) to bind to the pockets (Supplementary Figure S5C–F). Our docking analysis proved that the Pol tertiary structure can be used to screen potential drug candidates. Furthermore, the tertiary structure of HIV-1 Pol polyprotein is a great starting point to build the tertiary structure of full-length Gag-Pol polyprotein, which can be the ultimate study subject in autoprocessing.

There are multiple tools available for our prediction work and we tested the tools available and chose the most appropriate ones for us. For example, we selected GalaxyDomDock because it can return multiple models, instead of only one model from AIDA [62] and the top five models with similar conformations from DEMO [63]. The diverse conformations from GalaxyDomDock were required to determine the most reasonable model to form the dimer without clashes. Additionally, the reason we selected YASARA was that it is one of the top tools for structural optimization which can optimize the large-sized dimer structure. Not all tools can handle the protein dimer optimization in this large size. For example, ModRefiner [64] only returns a monomer structure even when the input is a dimer structure. The final model evaluation score for our predicted HIV-1 Pol polyprotein proves that the tools we used are appropriate and successful.

Finally, the workflow developed by this study can be used to construct the tertiary structure of other polyproteins that are difficult to resolve with traditional methods and for which the structure of the components are available, such as Gag/Pol/Gag-Pol protein from other retroviruses (e.g., HFV, MoMLV, FIV, MMTV, HTLV, and RSV) [28] and ORF1a/ORF1ab from coronaviruses (e.g., SARS-CoV, MERS-CoV, and SARS-CoV2) [29]. These polyproteins are precursors of structural proteins or enzymes of these viruses. The ability to construct their tertiary structures will further our understanding of the virus RNA and Gag/Gag-Pol assembly and maturation in the virus life cycle and will be very helpful to develop new classes of drugs which can interrupt the proteolytic cleavage of these polyproteins.

## 4. Materials and Methods

### 4.1. Protein Sequences, Structures, and Sequence Alignment

The HIV-1 strain NL4.3 genome sequenced from our lab [20] was used to obtain the corresponding Pol polyprotein sequence. The relevant protein structures used were obtained from the PDB database [1]: HIV-1 partial Pol structure (PDB ID: 7SJX) [13], HIV-1 RT structures including 4ZHR [38], 3E01 [39], 2JLE [40], 1DTT [41], 1C0U [42], 1C1B [43], and 1C1C [43], and HIV-1 IN structures including 1WJB [44], 1K6Y [45], 1WJA [44], 2ITG [46], 1B9D [47], 1ITG [48], 5HOT [49], and 1EX4 [50]. T-Coffee implemented in Jalview [36,37] was used for the sequence alignment.

### 4.2. Protein Structure Modeling, Optimization, and Domain Assembly Methods

Software tools and web applications used in this work for protein structure modeling, optimization, and domain assembly are listed in Table 1. We describe them briefly below.

SWISS-MODEL [30] was used to model the structure for a specific protein sequence. Herein, the “User Template Mode” option was used for the highly homologous proteins. Both the target sequence and template structure were used as the input. This method can predict the mutated residues or missing fragments within the structure. SWISS-MODEL [30] was also used to optimize the structure generated by other methods. The homodimer structure can also be modeled or optimized by using the single protein sequence and the corresponding homodimer structure as the template.

Robetta was used to predict the full-length protein structure with the comparative modeling (CM) method [31,32]. In the prediction task, 1000 models were sampled with all other parameters using default options (register shifts: 0; fragments: 0). The target protein sequence and template structures were used as the Robetta input. Of note, this process can build the missing structure in any region because Robetta predicts a missing fragment with a de novo technique.

In addition to the abovementioned structure modeling methods, an empirical strategy for constructing the complete protein structure was developed. Specifically, the “alter” and “create” commands implemented in Open-Source PyMOL (Table 1) were used to merge the small missing terminus fragment with the structure lacking this small missing fragment. The “alter” command was used to prepare the proper chain information for the two structures and the “create” command was used to merge the two separate structure objects into one structure object.

For the dimer structure construction, an anchor-based procedure was used as previously described [65]. Briefly, the target structure was aligned to the corresponding subunit of the anchor molecule. The anchor structure was then removed, and the remaining structure was used as the initial dimer structure. The initial structure was further optimized by either SWISS-MODEL as mentioned above or GalaxyRefineComplex [33]. For the GalaxyRefineComplex optimization, the initial dimer structure was used as the only input.

For the final Pol dimer optimization, YASARA Energy Minimization Server [35] was first used to perform large-scale energy minimization. The optimized structure was then extracted in PDB format by YASARA View.

Domain assembly refers to connecting two domains by linker(s). GalaxyDomDock [34] was used for performing the domain assembly task. Briefly, the “1-linker case” option was used to assemble two domains with one loop (i.e., linker). The whole protein sequence, structures of two domains, and the sequence ranges of domains and the linker were used as input. It required that the number of residues for each domain must be ≤600 and the linker be ≤30. The top 50 resulting models were returned.

GROMACS (version 2021) [51] was used to further evaluate the model stability. We used all-atom OPLS force field and the SPC/E water as the solvent. We performed 50,000 steps for energy minimization, and 300 ps equilibration for both NVT and NPT. Finally, we ran 5 ns production MD simulation for investigating the structural stability. Other parameter settings followed the protein in water simulation conditions [52].

### 4.3. Protein Model Quality Estimation

Computational protein simulation can be used as an alternative technique to help researchers generate the corresponding atomic model when experimental protein determination is difficult due to various reasons. However, the model quality should be evaluated. In this work, MolProbity score [53] implemented in SWISS-MODEL and a PISA-based radar plot [54] were used for estimating the quality of the final Pol dimer model.

## 5. Conclusions

In this study, we developed a computational workflow which was composed of a set of different software tools and web applications. Using this workflow, we successfully predicted the HIV-1 NL4.3 Pol polyprotein dimer structure with improved quality scores compared to the reference structure, 7SJX. To our knowledge, our HIV-1 NL4.3 Pol dimer model is the first full-length HIV-1 Pol tertiary structure. It can provide a structural subject for studying the autoprocessing mechanism of HIV-1 Pol and for developing novel drugs targeting this process, which is in great demand due to the quick development of resistance to the existing HIV-1 drugs. In addition, the structure of HIV Pol dimer will be the foundation to construct the structure of the full-length Gag-Pol dimer. Moreover, the workflow can be used to predict other large protein structures, such as Gag/Gag-Pol in other retroviruses and ORF1a and ORF1ab in coronaviruses, represented by three famous pathogens, SARS-CoV, MERS-CoV, and SARS-CoV2, which cannot be resolved by conventional methods (e.g., X-ray crystallography or cryo-EM) due to the limitations of these technologies.

## Figures and Tables

**Table 1:** Software tools/web applications for performing HIV-1 NL4.3 Pol dimer modeling.

Name	Version/Accession Date	URL	Representative Function	References
GalaxyDomDock	04-2023	https://galaxy.seoklab.org/cgi-bin/submit.cgi?type=DOMDOCK_INTRO	Domain assembly	[34]
SWISS-MODEL	01-2023	https://swissmodel.expasy.org/	Structural modeling/optimization	[30]
Robetta	01-2023	https://robetta.bakerlab.org/	Structural modeling	[31,32]
GalaxyRefineComplex	02-2023	https://galaxy.seoklab.org/cgi-bin/submit.cgi?type=COMPLEX	Structural optimization	[33]
YASARA Energy Minimization Server	08-2023	http://www.yasara.org/minimizationserver.htm	Structural optimization	[35]
YASARA View	v.23.5.19	http://www.yasara.org/viewdl.htm	YASARA file operation	[35]
Open-Source PyMOL	v.2.5.0	https://github.com/schrodinger/pymol-open-source	Structural modeling/visualization	-
Jalview	v.2.11.3.1	https://www.jalview.org/	Sequence alignment	[36,37]

**Table 2:** Comparison of PISA radar interface parameters between our modeled HIV-1 NL4.3 Pol dimer and published partial HIV-1 Pol of 7SJX.

PISA Radar Interface Parameters	Our Modeled Pol Dimer	7SJX
NSB	90%	58%
NHB	81%	94%
HYP	77%	84%
TBE	90%	97%
SOE	96%	96%
INA	96%	96%
NDB	22%	22%

## Data Availability

All data used in this study are listed in Section 4: Materials and Methods section. The predicted HIV-1 NL4.3 Pol dimer structure is provided in the Supplementary data.

## Supplementary Materials

The following supporting information can be downloaded at: https://www.mdpi.com/article/10.3390/ijms25031809/s1. References [60,61,66,67,68] are cited in the Supplementary Material.
