# Supplementary material for: Modeling and Analysis of HIV-1 Pol Polyprotein as a Case Study for Predicting Large Polyprotein Structures
Source: Int J Mol Sci. 2024 Feb 2;25(3):1809. doi: 10.3390/ijms25031809 (PMC10855158; doi:10.3390/ijms25031809)
Supplement: Supplementary file 1 [file ijms-25-01809-s001.zip › Supplementary Material.pdf]

Supplementary Material

Docking Analysis of Predicted NL4.3 HIV-1 Pol Polyprotein with Known Inhibitors

The docking tool, gnina [1,2], was used to perform the docking studies between our modeled NL4.3 HIV-1 Pol dimer as the receptor and three ligands including a PR inhibitor, darunavir (DRV) from PDB ID of 6OPS [3], an RT inhibitor (GW695634) from PDB ID of 3DOL [4] and an IN inhibitor (KF116) from PDB ID of 4O55 [5]. Before performing the docking analysis, we first performed the alignment between each reference PDB structure with the corresponding ligand to the Pol dimer model by assuming that the ligand could bind to the Pol structure.

For the PR inhibitor binding analysis, we extracted the DRV as the ligand and the Pol dimer as the receptor. The gnina parameters were listed as follows: the docking box based on the aligned DRV and exhaustiveness of 64.

For the RT and IN inhibitor docking experiments, we performed an additional step compared to the PR inhibitor, which was the optimization preprocessing for the aligned ligand and the corresponding Pol dimer structure by using YASARA. After optimization, the ligand and receptor formed a proper binding conformation, which is one of the important steps for successful docking studies.

The docking study results for these three small molecular inhibitors are shown in **Supplemental Figure 5**. As shown in **Supplemental Figures 5A, 5C and 5E**, the reference PDB structures align to the corresponding Pol domains (i.e., PR domain, RT domain and IN domain) well, separately. By performing this operation, we assumed that these inhibitors could bind to the immature Pol structure in a similar way as their mature proteins. Finally, we extracted the ligand and receptor separately from the aligned complex and performed the re-docking procedure using gnina. As shown in **Supplemental Figures 5B, 5D and 5F**, the predicted optimal binding poses for DRV, GW695634, and KF116 superimpose well to their reference crystal ligands with the RMSD values of 1.40 Å, 0.79 Å and 0.21 Å, respectively. Therefore, the modeled NL4.3 HIV-1 Pol dimer model could be used for the docking studies if the assumption mentioned above occurs. However, it should be emphasized that the topic of how to use the large immature polyprotein (e.g., the modeled Pol dimer here) for computational studies (e.g., protein-ligand docking, protein-protein docking, and protein mutation stability analysis) is still an open question and needs to be explored further.

Supplemental Figures:

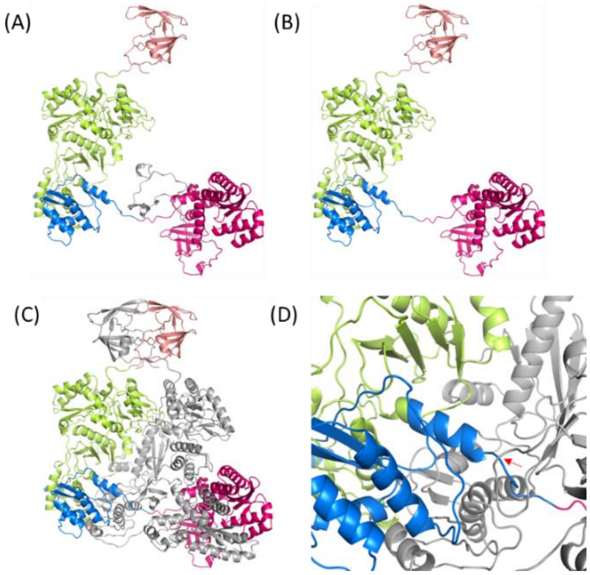

Supplementary Figure 1. Experimental process of modeling chain B in NL4.3 HIV-1 Pol. (A) Modeled chain B of NL4.3 HIV-1 Pol after adding 59 residues from IN CTD of chain A to IN CTD of chain B colored in gray, while the PR domain is colored in salmon, RT domain in limon, RH domain in marine and IN domain in hot pink. (B) Same as (A) but removing 59 residues from IN CTD of chain B. (C) Modeled NL4.3 HIV-1 Pol dimer by adding chain A of Pol colored in gray to chain B. (D) Enlarged (C) with the conflicting part highlighted by the red arrow.

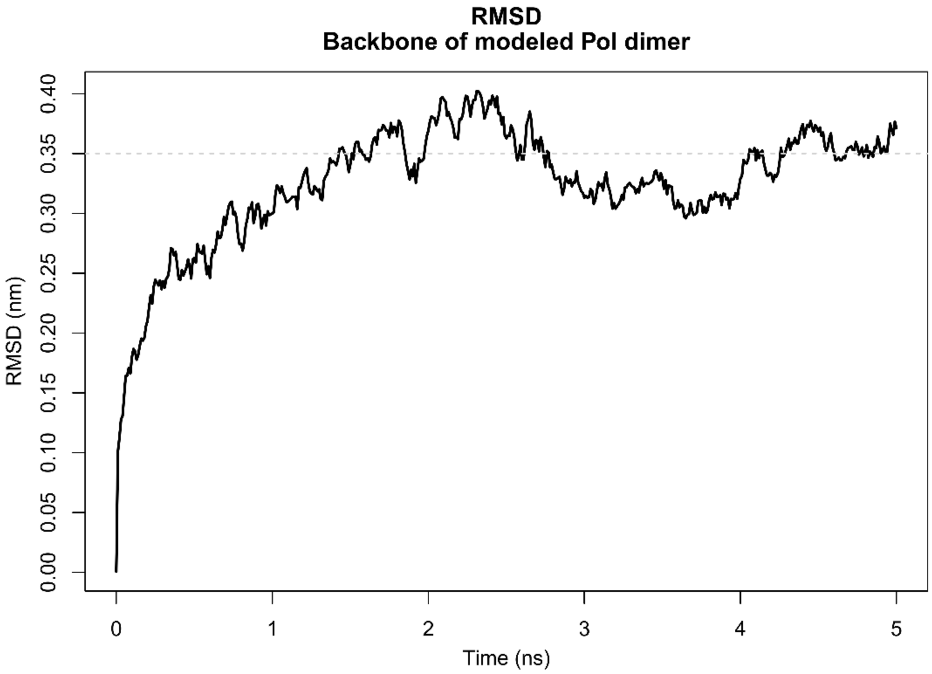

Supplementary Figure 2. Root-mean-square deviation (RMSD) of the modeled HIV-1 Pol dimer versus the simulation time of molecular dynamics.

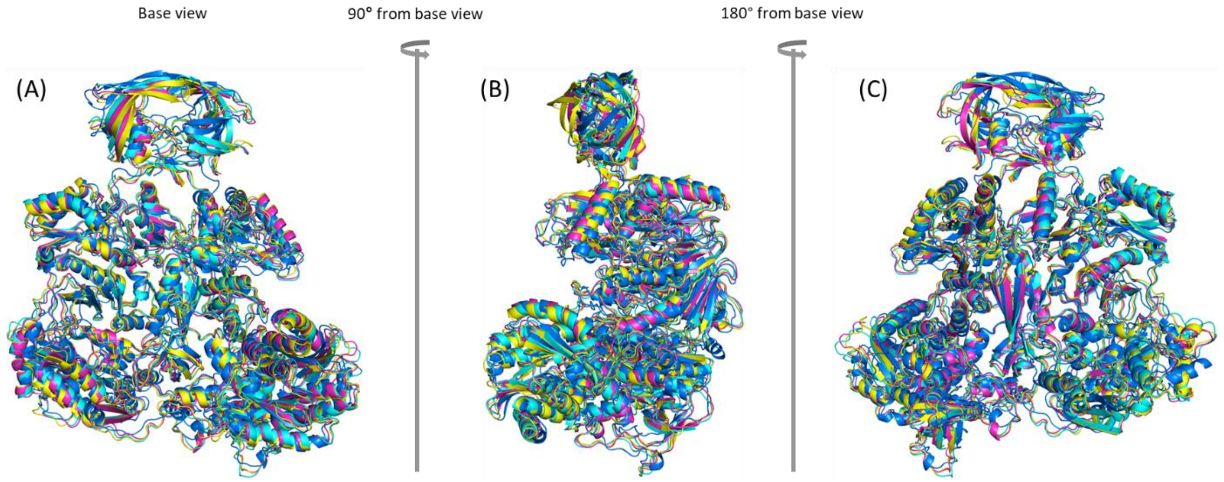

Supplementary Figure 3. Alignment of the original modeled HIV-1 Pol dimer, 4.8 ns-simulation structure, 4.9 ns simulation structure and 5 ns simulation structure. (A) The base view of the alignment, where the original modeled HIV-1 Pol dimer is colored in marine, 4.8 ns-simulation structure in cyan, 4.9 ns-simulation structure in magenta, and 5 ns-simulation structure in yellow.

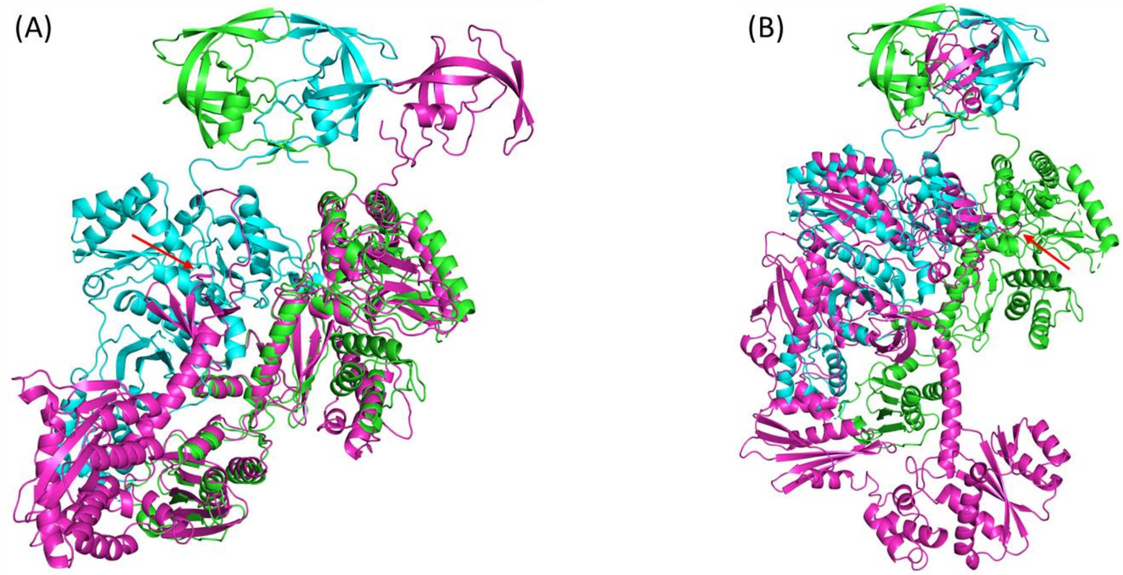

Supplementary Figure 4. Comparison of the AlphaFold2 modeled structure (magenta) with the reference structure, 7SJX (chain A: green; chain B: cyan). (A) Alignment of the AlphaFold2 modeled structure (magenta) to chain A (green) of 7SJX, where the PR domain from the AlphaFold2 model cannot align well with the PR domain of chain A from 7SJX and several conflicting parts with chain B of 7SJX can be found as shown by the red arrow. (B) Alignment of the AlphaFold2 modeled structure (magenta) to chain B (cyan) of 7SJX, where the PR domain from the AlphaFold2 model cannot align well with the PR domain of chain B from 7SJX and several conflicting parts with chain A of 7SJX can be found as indicated by the red arrow.

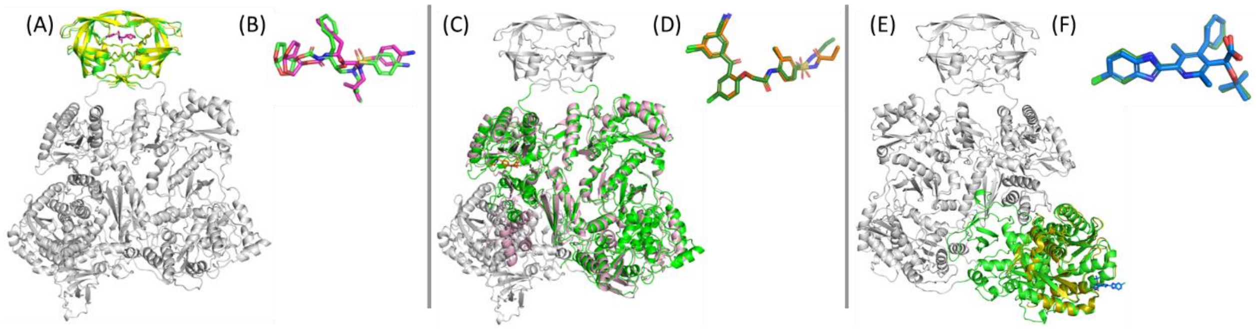

Supplementary Figure 5. Binding studies among PR inhibitor, RT inhibitor and IN inhibitor with the modeled HIV-1 Pol dimer. (A) Alignment between 6OPS (yellow) and the Pol PR domain (green), where other parts of Pol are colored in gray. (B) Alignment between the predicted optimal binding pose (C: green; O: red; N: blue; S: yellow) with the reference ligand DRV (C: magenta; O: red; N: blue; S: yellow) with RMSD of 1.40 Å. (C). Alignment between 3DOL (light pink) and the Pol RTp66 domain (green), where other parts of Pol are in gray. (D) Alignment between the predicted optimal binding pose (C: forest; O: red; N: blue; S: yellow; Cl: green) with the reference ligand GW695634 (C: orange; O: red; N: blue; S: yellow; Cl: green) with RMSD of 0.79 Å. (E) Alignment between 4O55 (olive) and the Pol IN domain (green), where other parts of Pol are in gray. (F) Alignment of the predicted optimal binding pose (C: forest; O: red; N: blue; Cl: green) with the reference ligand KF116 (C: marine; O: red; N: blue; Cl: green) with RMSD of 0.21 Å.

Reference

1. McNutt, A.T.; Francoeur, P.; Aggarwal, R.; Masuda, T.; Meli, R.; Ragoza, M.; Sunseri, J.; Koes, D.R. GNINA 1.0: molecular docking with deep learning. *J. Cheminform.* **2021**, *13*, 1-20.
2. Ragoza, M.; Hochuli, J.; Idrobo, E.; Sunseri, J.; Koes, D.R. Protein–ligand scoring with convolutional neural networks. *J. Chem. Inf. Model.* **2017**, *57*, 942-957.
3. Henes, M.; Lockbaum, G.J.; Kosovrasti, K.; Leidner, F.; Nachum, G.S.; Nalivaika, E.A.; Lee, S.-K.; Spielvogel, E.; Zhou, S.; Swanstrom, R. Picomolar to micromolar: elucidating the role of distal mutations in HIV-1 protease in conferring drug resistance. *ACS Chem. Biol.* **2019**, *14*, 2441-2452.
4. Ren, J.; Chamberlain, P.P.; Stamp, A.; Short, S.A.; Weaver, K.L.; Romines, K.R.; Hazen, R.; Freeman, A.; Ferris, R.G.; Andrews, C.W. Structural basis for the improved drug resistance profile of new generation benzophenone non-nucleoside HIV-1 reverse transcriptase inhibitors. *J. Med. Chem.* **2008**, *51*, 5000-5008.
5. Sharma, A.; Slaughter, A.; Jena, N.; Feng, L.; Kessl, J.J.; Fadel, H.J.; Malani, N.; Male, F.; Wu, L.; Poeschla, E. A new class of multimerization selective inhibitors of HIV-1 integrase. *PLoS Pathog.* **2014**, *10*, e1004171.
